# Renal Resistive Index Is Associated With Inactive Matrix Gla (γ‐Carboxyglutamate) Protein in an Adult Population‐Based Study

**DOI:** 10.1161/JAHA.119.013558

**Published:** 2019-09-12

**Authors:** David A. Jaques, Edward Pivin, Menno Pruijm, Daniel Ackermann, Idris Guessous, Georg Ehret, Fang‐Fei Wei, Jan A. Staessen, Antoinette Pechère‐Bertschi, Cees Vermeer, Bruno Vogt, Michel Burnier, Pierre‐Yves Martin, Murielle Bochud, Belen Ponte

**Affiliations:** ^1^ Division of Nephrology Geneva University Hospitals Geneva Switzerland; ^2^ Division of Chronic Disease University Institute of Social and Preventive Medicine Lausanne University Hospital Lausanne Switzerland; ^3^ Division of Nephrology and Hypertension Lausanne University Hospital and University of Lausanne Lausanne Switzerland; ^4^ University Clinic for Nephrology, Hypertension and Clinical Pharmacology Bern University Hospital Bern Switzerland; ^5^ Division of Primary Care Medicine Department of Primary Care Medicine Geneva University Hospitals Geneva Switzerland; ^6^ Division of Cardiology Geneva University Hospitals Geneva Switzerland; ^7^ Studies Coordinating Centre Research Unit of Hypertension and Cardiovascular Epidemiology KU Leuven Department of Cardiovascular Sciences University of Leuven Belgium; ^8^ R&D Group VitaK Maastricht University Maastricht The Netherlands; ^9^ Division of Hypertension Geneva University Hospitals Geneva Switzerland

**Keywords:** atherosclerosis, matrix Gla (γ‐carboxyglutamate) protein, pulse pressure, pulse wave velocity, renal physiology, renal resistive index, vascular stiffness, Biomarkers, Ultrasound, Atherosclerosis, Cardiovascular Disease, Nephrology and Kidney

## Abstract

**Background:**

Increased renal resistive index (RRI) has been associated with target organ damage as well as renal and cardiovascular outcomes. Matrix Gla (γ‐carboxyglutamate) protein (MGP) is a strong inhibitor of soft tissue calcification. Its inactive form (dephospho‐uncarboxylated MGP [dp‐ucMGP]) has been associated with vascular stiffness, cardiovascular outcomes, and mortality. In this study, we hypothesized that high levels of dp‐ucMGP were associated with increased RRI.

**Methods and Results:**

We recruited participants via a multicenter family‐based cross‐sectional study in Switzerland. Levels of dp‐ucMGP were measured in plasma by sandwich ELISA. RRI was measured by Doppler ultrasound in 3 segmental arteries in both kidneys. We used mixed regression models to assess the relationship between dp‐ucMGP and RRI. We adjusted for common determinants of RRI as well as renal function and cardiovascular risk factors. We included 1006 participants in our analyses: 526 women and 480 men. Mean values were 0.44±0.20 nmol/L for dp‐ucMGP and 64±5% for RRI. After multivariable adjustment, dp‐ucMGP was positively associated with RRI (*P*=0.001). In subgroup analysis by age tertiles, this association was not significant in the youngest age group (<38 years; *P*=0.62), whereas it was significant in older age groups (38–55 and >55 years; *P*=0.016 and *P*<0.001, respectively).

**Conclusions:**

Levels of dp‐ucMGP are positively and independently associated with RRI after adjustment for common determinants of RRI, cardiovascular risk factors, and renal function. The stronger association among older adults is probably due, in part, to age‐related arterial stiffness. RRI thus seems to reflect the global atherosclerotic burden in a general adult population.

**Clinical Perspective**

**What Is New?**

- We assessed the relationship between renal resistive index and the inactive form of matrix Gla (γ‐carboxyglutamate) protein (dephospho‐uncarboxylated matrix Gla protein) as a marker of systemic vascular calcification in a general adult population.
- Renal resistive index was positively associated with levels of dephospho‐uncarboxylated matrix Gla protein, independent of common determinants of renal resistive index, cardiovascular risk factors, and renal function.

**What Are the Clinical Implications?**

- These findings suggest that renal resistive index reflects the global atherosclerotic burden in healthy participants and could identify patients who might benefit from vitamin K supplementation.
- Whether this supplementation would result in a meaningful improvement in clinical outcome remains to be demonstrated in randomized controlled trials.

## Introduction

Renal resistive index (RRI) is the difference between the maximum systolic velocity and the end‐diastolic velocity divided by the maximum systolic velocity. Velocities are measured by renal Doppler ultrasound of interlobar arteries.^1^ Despite use for several decades, the clinical interpretation of RRI remains unclear. In a population‐based study that included people with mainly preserved renal function and normal renal ultrasound, factors associated with RRI were sex, age, blood pressure (BP), heart rate (HR), and body mass index (BMI).^2^ In patients with kidney disease, RRI correlated with the renal pathological process itself.^3^ In contrast, in kidney transplant recipients, RRI depended mainly on systemic vascular properties.^4^ Moreover, an elevated RRI could serve as a marker of target organ damage, as RRI is associated with adverse renal and cardiovascular outcomes.^5^ This latter finding supports the hypothesis that intrinsic renal resistance is only a minor determinant of RRI and that RRI depends mainly on extrarenal systemic hemodynamic parameters such as pulse pressure (PP) and vascular compliance.^6^ High PP and/or low vascular compliance would thus be the main predictors of elevated RRI. This theoretical view is supported by some experimental evidence, but information from clinical studies in humans remains sparse.^7^, ^8^, ^9^

Matrix Gla (γ‐carboxyglutamate) protein (MGP) is a strong inhibitor of soft tissue calcification.^10^, ^11^ MGP knockout mice develop massive vascular calcification in their first weeks of life and die within 2 months of vessel rupture.^12^ To acquire its full calcification inhibitory activity, MGP needs to undergo 2 posttranslational modifications. Consequently, 4 different MGP conformations can be found: (1) unmodified and inactive dephospho‐uncarboxylated MGP (dp‐ucMGP), (2) phosphorylated only, (3) carboxylated only, and (4) fully modified and active phosphorylated and carboxylated MGP.^13^, ^14^ Carboxylation of MGP is a vitamin K–dependent process because vitamin K hydroquinone is an active cofactor of the carboxylation of glutamate residues into Gla.^15^ Therefore, dp‐ucMGP is an inverse marker of vitamin K status, in which high circulating levels reflect poor vitamin K stores.^16^ Active MGP adsorbs extracellular hydroxyapatite, inhibiting crystal growth.^15^ It also inhibits vascular smooth muscle cell apoptosis by binding to BMP2 (bone morphogenetic protein 2), a proapoptotic agonist.^17^ Active MGP protects the microcirculation and organ function.^18^ Levels of dp‐ucMGP have been associated with vascular stiffness measured by carotid–femoral pulse wave velocity (PWV) in 2 population‐based independent studies involving >1000 participants.^19^, ^20^

To clarify the pathophysiological and clinical meaning of RRI in the general adult population, we assessed whether RRI was associated with dp‐ucMGP as a marker of systemic vascular calcification.

## Methods

The data that support the findings of this study are available from the corresponding author upon reasonable request.

### Participant Selection

SKIPOGH (Swiss Kidney Project on Genes in Hypertension) is a family‐based cross‐sectional study exploring the role of genes and kidney hemodynamics in BP regulation and kidney function in the general population.^21^ From December 2009 to March 2013, adult participants were recruited in 2 regions (Bern and Geneva) and 1 city (Lausanne) in Switzerland. The population‐sampling method has been described in detail previously.^21^ Inclusion criteria were (1) a minimum of 18 years of age, (2) European ancestry, (3) first‐degree family members who were willing to participate, and (4) written informed consent. Pregnant or breastfeeding women were not included. The institutional ethics committees of the participating university hospitals approved the SKIPOGH study.

### Study Visit

Participants were seen in the morning after an overnight fast. BP was measured 5 times after a resting period of ≥10 minutes in the sitting position with a nonmercury manual auscultatory sphygmomanometer (A&D UM‐101), according to the guidelines of the European Society of Hypertension.^22^ For the analyses, we used the mean of the last 4 BP and HR measurements.

### Laboratory Data

Blood venous samples were drawn after an overnight fast. Blood glucose, electrolytes, kidney and liver function tests, serum cholesterol, and triglycerides were analyzed by standard clinical laboratory methods. Levels of dp‐ucMGP were assessed in EDTA plasma using the InaKtif iSYS Kit (IDS), which is a precommercial dual‐antibody test based on the sandwich ELISA, developed by VitaK (Maastricht University, The Netherlands). Intra‐assay variation coefficients are 3.1% for the upper limit of the normal range and 5.4% for the lower limit of the normal range. Interassay variation coefficients are 6.9% for the upper limit of the normal range and 13.6% for the lower limit of the normal range (Unpublished data from Cees Vermeer, PhD, R&D Group VitaK, Maastricht University, Maastricht, The Netherlands).

### Renal Doppler Ultrasound

In each study center, the same experienced operator performed renal gray‐scale and color duplex ultrasounds, according to a standardized procedure, as described previously.^2^ Briefly, RRI was measured in 3 segmental arteries (superior, middle, and inferior) of each kidney. The values were then averaged to obtain a mean value for each participant. The reproducibility of RRI measurements was assessed in a subgroup of 20 unrelated participants with inter‐ and intraobserver Lin correlation coefficients of 0.75 and 0.89, respectively, for the right kidney and 0.69 and 0.72, respectively, for the left kidney. Values were multiplied by 100 to express the RRI results as percentages.

### Pulse Wave Velocity

Arterial waveforms were assessed during an 8‐second period in the supine position after 15 minutes of rest at the carotid and femoral arteries by applanation tonometry, using a high‐fidelity SPC‐301 micromanometer (Millar Instruments), interfaced with a laptop computer running the SphygmoCor software v8.0 or v8.2 (AtCor Medical). In each study center, the same experienced operator obtained the arterial waveforms and recorded carotid–femoral PWV, as described previously.^19^

### Definitions

Diabetes mellitus was defined as present when reported or treated or when fasting blood glucose level was ≥7 mmol/L. Hypertension was considered present when treated or when mean office BP was ≥140/90 mm Hg, according to European guidelines.^22^ PP was defined as systolic BP (SBP) minus diastolic BP (DBP). The Chronic Kidney Disease Epidemiology Collaboration (CKD‐EPI) formula was used to estimate glomerular filtration rate (eGFR), and an eGFR <60 mL/min per 1.73 m^2^ defined chronic kidney disease. Kidney ultrasound was considered abnormal in any case of unilateral kidney, cysts, atrophy, stone, hydronephrosis, partial nephrectomy, suspected renal artery stenosis, horseshoe kidney, or any other kidney malformation.

### Statistical Analysis

Continuous variables are expressed as mean±SD and categorical variables as number and relative frequencies. Normality of distribution was assessed graphically. Outliers were defined as participants with RRI or dp‐ucMGP values >99th or <1st percentile. RRI was divided in tertiles to assess associations with baseline characteristics. Variables were compared using χ^2^ tests and ANOVA or Kruskal–Wallis tests (depending on normality and homoscedasticity) for categorical and continuous variables, respectively, and Cuzick trend tests.

In univariable linear regression analysis, we explored the relationship of RRI with the following variables: dp‐ucMGP, age, height, BMI, HR, eGFR, LDL (low‐density lipoprotein), HDL (high‐density lipoprotein), glucose, SBP, DBP, sex, smoking status, diabetes mellitus, antihypertensive treatment, dyslipidemia treatment, and cardiovascular disease (CVD).

To characterize the relationship between RRI and dp‐ucMGP, multiple linear regressions were conducted using a backward stepwise approach, and in the final model, only variables with *P*<0.05 were kept. In a first step, variables associated with RRI were identified, omitting dp‐ucMGP. Similarly, variables associated with dp‐ucMGP were identified, omitting RRI. Then we set a model including as covariates all variables yielded from the previous 2 steps, with dp‐ucMGP as the factor of interest and RRI as the outcome. Because age has been demonstrated to have a quadratic relationship with RRI, age and the square of age were included in multivariable models.^2^ All models were treated as mixed models with the participant's family as the clustering variable and intercept as a random effect. All models were also adjusted for study center as a fixed effect. Results are presented as β coefficients (corresponding to a 1‐U increment for continuous variables or difference between 2 categories in categorical variables) and associated 95% CIs and *P* values. An alternative analysis used mixed multivariable logistic regression with dp‐ucMGP >70% as the dependent variable. The other characteristics of this model were identical to our main linear model.

In the final multivariable model, we looked for a modification effect or interaction between dp‐ucMGP and the following variables: abnormal kidney on ultrasound, sex, chronic kidney disease, antihypertensive treatment, hypertension, and age. Because age has a quadratic relationship with RRI, this variable was divided in tertiles to allow interaction testing. The likelihood ratio test (LRT) was used to compare models with and without interaction terms. Linearity was assessed graphically by scatterplots and by LRT comparing models including the variable as continuous versus categorical.

As secondary analyses, we explored the relationship among RRI, PP, and PWV. In a first multivariable regression, only PP and PWV were considered as independent variables. In a second multivariable regression model, factors associated with RRI in our main model (except dp‐ucMGP) were introduced as independent variables.

Data were considered to be missing completely at random; therefore, patients with any missing variables were excluded from the multivariable analyses. Statistical analyses were conducted using STATA v15 (StataCorp). *P*<0.05 was considered significant. For interactions, the *P* value was set at <0.1 for interaction terms and/or LRT.

## Results

The complete SKIPOGH cohort included 1128 participants. For these analyses, we excluded 4 participants on anti–vitamin K medication and 80 with missing data on RRI and/or dp‐ucMGP. Among the 1044 remaining participants, 38 were outliers, leaving 1006 for the main analyses (Figure S1).

Mean values of RRI and dp‐ucMGP were 64±5% and 0.44±0.20 nmol/L, respectively. In total, 128 of 1006 patients (12.7%) had RRI values >70%. Overall, 52.3% were women, and mean age was 46.7±17.2 years. Participant characteristics are described according to RRI tertiles in Table 1. Low, medium, and high RRI tertile values were <61%, 61% to 65% and >65%, respectively. Women had higher RRIs than men (*P*<0.001). Across increasing tertiles of RRI, more participants had diabetes mellitus, hypertension, chronic kidney disease, history of cardiovascular disease, BP‐ and lipid‐lowering drugs, or abnormality on kidney ultrasound (all *P*<0.05). There was a trend only toward fewer smokers in higher RRI tertiles. Dp‐ucMGP, PWV, age, BMI, glucose, and SBP increased within RRI tertiles (all *P*<0.001), whereas height, eGFR, and DBP decreased with higher categories of RRI (all *P*<0.05). There was a trend only toward higher LDL and fewer smokers across increasing tertiles (for trend, *P*=0.04 and *P*=0.03, respectively).

**Table 1:** Patients Characteristics According to Tertiles of RRI (n=1006, Without Outliers)

Characteristics	n	Overall	Low RRI (<61%) n=347	Medium RRI (61–65%) n=333	High RRI (>65%) n=326	*P* Value
RRI mean value %	1006	64±5	58±2	63±1	70±3	<0.001
Categorical variables and comorbidities, n (%)						
Female	1006	526 (52.3)	151 (43.5)	177 (53.2)	198 (60.7)	<0.001
Smoker	1006	243 (24.2)	94 (27.1)	84 (25.2)	65 (19.9)	0.08^a^
Diabetes mellitus	996	34 (3.4)	3 (0.9)	5 (1.5)	26 (8.1)	<0.001
Hypertension	1002	222 (22.2)	42 (12.2)	50 (15)	130 (40.1)	<0.001
BP‐lowering drugs	1003	151 (15.1)	23 (6.7)	34 (10.2)	94 (28.9)	<0.001
Lipid‐lowering drugs	1003	39 (3.9)	4 (1.2)	17 (5.1)	18 (5.5)	0.01
CVD	994	99 (10.0)	16 (4.7)	34 (10.2)	49 (15.4)	<0.001
Abnormal kidney	999	189 (18.9)	40 (11.7)	67 (20.2)	82 (25.2)	<0.001
CKD	1006	23 (2.3)	3 (0.9)	1 (0.3)	19 (5.8)	<0.001
Clinical characteristics, mean±SD						
Age, y	1006	46.7±17.2	38.6±13.0	44.2±15.2	58.1±17.1	<0.001
Height, cm	1006	171±9	173±9	171±9	167±9	<0.001
BMI, kg/m^2^	1006	25.0±4.5	24.0±3.8	25.2±5.0	25.8±4.5	<0.001
SBP, mm Hg	1005	117±16	112±12	114±14	125±20	<0.001
DBP, mm Hg	1005	75.5±9.7	76.6±9.6	74.8±9.2	75.1±10.3	0.04
PP, mm Hg	1005	41.5±11.8	35.8±7.5	39.5±8.5	49.7±13.7	<0.001
HR, beats/min	999	66.7±10.7	67.6±11.0	66.1±10.7	66.2±10.2	0.1
Laboratory characteristics, mean±SD						
dp‐ucMGP, nmol/L	1006	0.44±0.20	0.39±0.17	0.43±0.20	0.52±0.22	<0.001
GFR, mL/min/1.73 m^2^	1000	97.2±17.3	104±15	99.1±15.2	88.1±18.0	<0.001
LDL, mmol/L	995	3.11±0.92	3.05±0.92	3.11±0.90	3.19±0.94	0.12^b^
HDL, mmol/L	999	1.51±0.42	1.50±0.41	1.51±0.42	1.51±0.43	0.98
Glucose, mmol/L	1000	5.16±0.72	4.96±0.58	5.08±0.55	5.46±0.90	<0.001
PWV, m/s	923	7.90±2.22	7.14±1.37	7.53±1.82	9.08±2.76	<0.001

BMI indicates body mass index; BP, blood pressure; CKD, chronic kidney disease; CVD, cardiovascular disease; DBP, diastolic blood pressure; dp‐ucMGP, dephospho‐uncarboxylated matrix Gla (γ‐carboxyglutamate) protein; GFR, glomerular filtration rate; HDL, high‐density lipoprotein; HR, heart rate; LDL, low‐density lipoprotein; PP, pulse pressure; PWV, pulse wave velocity; RRI, renal resistive index; SBP, systolic blood pressure.

a *P*=0.03 for trend.

b *P*=0.04 for trend.

### Association of dp‐ucMGP With RRI

**Figure 1:**
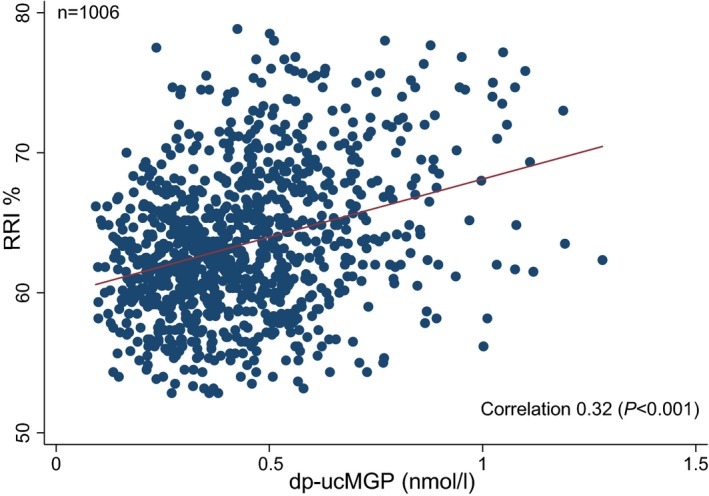
Scatterplot showing the association between dp‐ucMGP and RRI (n=1006, without outliers). Red line represents linear regression. Pearson correlation coefficient is represented with the corresponding *P* value. dp‐ucMGP indicates dephospho‐uncarboxylated matrix Gla (γ‐carboxyglutamate) protein; RRI, renal resistive index.

In univariable analyses, all independent variables except LDL, HDL, and DBP were associated with RRI (Table 2). A positive association of dp‐ucMGP with RRI was noted (Figure 1): every 1‐U increase in dp‐ucMGP (nmol/L) increased RRI by 8.1%.

**Table 2:** Factors Associated With RRI in Univariable and Multivariable Mixed Linear Regression Models (n=970 for All Models, Without Outliers)

Independent Variable	Univariable Model			Full Model			Final Model		
	β	95% CI	*P* Value	β	95% CI	*P* Value	β	95% CI	*P* Value
dp‐ucMGP, nmol/L	8.06	6.60–9.53	<0.001	2.17	0.96–3.42	0.001	2.18	0.96–3.41	0.001
Sex (female)	1.63	1.03–2.22	<0.001	1.88	1.19–2.57	<0.001	1.73	1.27–2.18	<0.001
Smoker (yes)	−1.08	−1.80 to −0.37	0.003	0.29	−0.21 to 0.79	0.26	···	···	···
Diabetes mellitus (yes)	6.24	4.62–7.87	<0.001	0.64	−0.71 to 1.98	0.35	···	···	···
BP drugs (yes)	4.60	3.79–5.41	<0.001	0.19	−0.49 to 0.87	0.59	···	···	···
Lipid drugs (yes)	2.54	0.93–4.16	0.002	−0.55	−1.68 to 0.59	0.35	···	···	···
CVD (yes)	3.95	2.96–4.93	<0.001	0.51	−0.23 to 1.24	0.18	···	···	···
Age, y	−0.33	−0.40 to −0.26	<0.001	−0.15	−0.23 to −0.07	<0.001	−0.16	−0.23 to −0.08	<0.001
Age^2^, y	0.01	0.004–0.006	<0.001	0.003	0.002–0.003	<0.001	0.003	0.002–0.004	<0.001
Body height, cm	−0.17	−0.20 to −0.14	<0.001	−0.01	−0.04 to 0.03	0.79	···	···	···
BMI, kg/m^2^	0.19	0.12–0.26	<0.001	0.06	0.00–0.12	0.049	0.08	0.02–0.14	0.006
SBP, mm Hg	0.11	0.09–0.13	<0.001	0.12	0.10–0.15	<0.001	0.13	0.10–0.15	<0.001
DBP, mm Hg	−0.02	−0.06 to 0.01	0.15	−0.22	−0.25 to −0.18	<0.001	−0.22	−0.26 to −0.18	<0.001
HR, beats/min	−0.06	−0.09 to −0.03	<0.001	−0.05	−0.07 to −0.03	<0.001	−0.05	−0.07 to −0.03	<0.001
GFR, mL/min/1.73 m^2^	−0.12	−0.13 to −0.10	<0.001	−0.001	−0.02 to 0.02	0.95	···	···	···
LDL, mmol/L	0.15	−0.19 to 0.48	0.39	−0.25	−0.51 to 0.01	0.06	−0.29	−0.54 to −0.04	0.025
HDL, mmol/L	0.24	−0.50 to 0.99	0.52	−0.58	−1.20 to 0.04	0.07	···	···	···
Glucose, mmol/L	1.98	1.55–2.42	<0.001	0.58	0.19–0.99	0.07	0.66	0.30–1.02	<0.001

All models are adjusted for center as fixed effect and for family as random effect. β corresponds to 1‐U increase. BMI indicates body mass index; BP, blood pressure; CVD, cardiovascular disease; DBP, diastolic blood pressure; dp‐ucMGP, dephospho‐uncarboxylated matrix Gla (γ‐carboxyglutamate) protein; GFR, glomerular filtration rate; HDL, high density lipoprotein; HR, heart rate; LDL, low density lipoprotein; RRI, renal resistive index; SBP, systolic blood pressure.

Multivariable analyses included 970 patients without missing data (Table 2). Factors associated with RRI were dp‐ucMGP, sex, age, age squared, BMI, HR, LDL, glucose, SBP, and DBP (all *P*<0.05). In the final model, every 1‐U increase in dp‐ucMGP (nmol/L) was associated with an increase of 2.2% RRI. During the backward stepwise procedure, variables were discarded in the following order: eGFR, diabetes mellitus, smoking, and history of CVD (Table S1). The following variables were discarded from the outset and thus not considered in the backward stepwise procedure, as they were not associated with either RRI or dp‐ucMGP in initial multivariable models (see Methods): height, HDL, BP, and lipid‐lowering drugs.

In multivariable logistic regression, dp‐ucMGP was significantly associated with RRI >70% (coefficient: 2.59; 95% CI, 1.11–4.08; *P*=0.001).

### Sensitivity and Secondary Analyses

**Figure 2:**
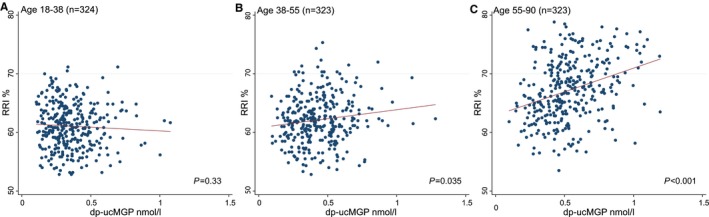
Scatterplots showing the univariable association between dp‐ucMGP and RRI depending on tertiles of age (n=970, without outliers and no missing covariates). **A**, Younger tertile (first): β=−1.1 (95% CI, −3.3 to +1.1). **B**, Middle tertile (second): β=2.4 (95% CI, 0.2–4.7). **C**, Older tertile (third): β=7.6 (95% CI, 5.1–10.0). Univariable *P* value for interaction <0.001. Red line represents linear regression line. *P* values from the regression are represented. dp‐ucMGP indicates dephospho‐uncarboxylated matrix Gla (γ‐carboxyglutamate) protein; RRI, renal resistive index.

In sensitivity analyses, results were globally similar when accounting for outliers (Table S2). Moreover, no interaction was found between dp‐ucMGP and abnormal kidney on ultrasound, sex, chronic kidney disease, antihypertensive treatment, and hypertension (interaction term, *P*>0.1; LRT, *P*>0.1 for all). However, a significant interaction was found between dp‐ucMGP and age in tertiles (*P*<0.001 for interaction term and LRT). The strength and significance of the association between dp‐ucMGP and RRI increased across tertiles of age (Figure 2). In the multivariable analysis stratified by age category, there was no significant association in the youngest group (aged 18–38 years: β=−0.53; 95% CI, −2.62 to 1.56; *P*=0.62), whereas the association was significant in the older groups (aged 38–55 years: β=2.58; 95% CI, 0.47–4.48; *P*=0.016; aged ≥55 years: β=4.62; 95% CI, 2.53–6.72; *P*<0.001).

In secondary analyses, PP and PWV showed a positive relationship with RRI (Pearson correlation coefficients of 0.59 and 0.45, respectively). However, graphical assessment showed nonlinearity, specifically, at the lowest PP and PWV values (Figure S2). Because nonlinearity was confirmed by LRT, PP and PWV were used as tertiles in linear regressions. In a mixed unadjusted linear regression model, PP and PWV were both positively associated with RRI (both *P*<0.001). PP and PWV were still significantly associated with RRI after adjustment for sex, age, BMI, HR, LDL, and glucose (*P*<0.001 and *P*=0.040, respectively).

## Discussion

In this study we observed a positive association between RRI and dp‐ucMGP in a large, multicentric, family‐based population. This association was independent of other variables previously known to influence RRI (age, sex, BMI, HR, SBP, and DBP) and renal function and cardiovascular risk factors (diabetes mellitus, treated hypertension, and smoking).

### MGP as a Determinant of RRI

Soon after being introduced in 1974 as a measure of intrarenal vascular resistance, RRI was used as a potential marker of kidney damage. Indeed, RRI theoretically depends on the capillary wedge pressure, representing a combination of interstitial and venous pressures.^6^ Any pathological process affecting one of these compartments should increase RRI. This was illustrated for the first time in 1991 in kidney biopsies, as tubulointerstitial disease was associated with higher RRI than isolated glomerulopathies.^23^ A larger study including 992 patients with simultaneous RRI assessment and kidney biopsy also showed that tubulointerstitial injury correlated strongly with elevated RRI.^3^ Consequently, in patients with established kidney injury, an elevated RRI reflects—at least in part—the intrarenal pathological process itself. In contrast, RRI could also reflect systemic vascular properties rather than intrarenal pathological processes. The most convincing data supporting this hypothesis thus far came from an elegant study of 321 renal transplant recipients.^4^ In that study, RRI correlated with recipient age and central hemodynamic factors but not with histopathological findings on protocol‐specified kidney biopsies. However, because that study took place in a specific population of transplanted patients, its generalizability is limited. The use of calcineurin inhibitors, known to influence the RRI, also hampered definite conclusions. The clinical interpretation of RRI is further complicated by the fact that RRI can also be seen as a marker of target kidney damage and a prognostic predictor of renal and cardiovascular outcome. Therefore, RRI was associated with albuminuria and left ventricular hypertrophy in 211 untreated hypertensive patients.^24^ Moreover, RRI was predictive of a composite end point of cardiovascular and renal events in 426 hypertensive patients followed for a mean of 3.1 years.^5^ Conversely, dp‐ucMGP was previously associated with various cardiovascular outcomes in several populations: high dp‐ucMGP levels were associated with below‐knee arterial calcification scores in patients with type 2 diabetes mellitus and an increased risk of CVD independent of classical risk factors in older patients free of CVD at baseline.^25^, ^26^ Moreover, dp‐ucMGP has already been associated with vascular calcification and arterial stiffness.^19^, ^20^, ^27^, ^28^, ^29^

Our study provides new data from the general population. The robust positive association of RRI with dp‐ucMGP described in our study links RRI, for the first time, to a direct pathophysiological marker of vascular calcification. Our findings support the view that RRI is a marker of systemic atherosclerotic burden. This hypothesis is reinforced by the fact that the association between RRI and dp‐ucMGP was independent of classical determinants of RRI (age, sex, BMI, HR) and, more important, from SBP and DBP. This finding further supports the idea that RRI reflects the global vascular calcification burden itself and not only systemic hemodynamic properties. Moreover, in our study, the association between RRI and dp‐ucMGP was not influenced by the presence of established CVD, traditional cardiovascular risk factors (diabetes mellitus, hypertension, and smoking), renal function, and the presence of abnormality on renal ultrasound. This highlights the idea that, in a general adult population, RRI reflects global vascular calcification status despite the absence of renal and cardiovascular clinical manifestations. Globally, our findings shed new light on the intricate role of RRI as a prognostic marker of renal and cardiovascular outcomes.

Finally, age seemed to play a complex role in the relationship between dp‐ucMGP and RRI. The effect and significance of dp‐ucMGP on RRI increased with advancing age. However, this result is not surprising because the range of absolute dp‐ucMGP values was low in younger people and thus less likely to reflect significant vascular hemodynamic impairment. Moreover, arterial stiffness is also known to increase with age.

### Determinants of RRI

Mathematical derivations also support the hypothesis that RRI depends on systemic vascular and hemodynamic factors. O'Neill stated that the RRI equation can be rewritten in the following fashion^6^: $\text{RRI}=1-\frac{\text{Pdia}-\text{P0}}{\text{Psys}-\text{P0}}\times \frac{\text{LAsys}}{\text{LAdia}}$, where Psys indicates systolic pressure, Pdia indicates diastolic pressure, P0 indicates renal capillary wedge pressure, LAsys indicates systolic vessel lumen area, and LAdia indicates diastolic vessel lumen area. In most physiological situations, P0 (interstitial plus venous pressure) is negligible and RRI indirectly depends on 2 central hemodynamic parameters: PP (represented here by the ratio of Pdia/Psys) and vascular compliance (represented here by the ratio of LAsys/LAdia). RRI thus varies directly with PP and inversely with vascular compliance. This interpretation of RRI sheds light on its dependency on extrarenal hemodynamic factors such as age, HR, and Valsalva maneuver.^30^, ^31^, ^32^ Experimental data support this theoretical view. First, RRIs of perfused rabbit kidneys showed a strong linear correlation with PP.^8^ Second, Hashimoto and Ito^7^ showed that RRI was dependent on aortic PP measured by applanation tonometry and aortic compliance measured by carotid–femoral PWV in 133 hypertensive patients. However, when using multivariable analysis, only aortic PP (not aortic compliance) was independently associated with RRI, suggesting that PP was the main physiological factor. Our findings globally agree with this model. Even after accounting for a nonlinear relationship, PP measured by manual auscultatory sphygmomanometer and aortic compliance measured by carotid–femoral PWV both showed a strong positive correlation with RRI. However, PWV seemed to play a weaker role than PP; its univariable correlation with RRI was smaller, and its statistical significance in multivariable analysis decreased substantially. This result again supports the fact that PP is an independent determinant of RRI, whereas aortic compliance could merely represent a secondary effect on the causal process. Globally, our findings confirm that in a healthy adult population, RRI is strongly dependent on extrarenal systemic hemodynamic factors.

### Limitations

The main limitation of our study is the observational and cross‐sectional nature of the association between RRI and dp‐ucMGP, which limits causal inference. Moreover, despite adjusting for clinically relevant variables, including known confounders, we cannot exclude potential residual confounding. RRI measurements were performed in 3 different study centers. However, the center effect was accounted for in linear regressions, and interobservator reproducibility was excellent. Strengths of our study include the large sample size with >1000 participants and the population‐based design, which increases its generalizability. However, this study included people of European descent, and whether similar associations exist in non‐European populations remains an open question. RRI could be influenced by intra‐abdominal pressure, hematoma, or cirrhosis, but because mainly healthy participants were included, we do not believe those conditions would significantly influence our results. Moreover, all measurements were performed in expiration, which reduces the influence of intra‐abdominal pressure. RRI also depends on stroke volume, which was not measured in our study. However, there is no physiological argument that adjustment for this parameter would alter the strength or direction of the association between RRI and dp‐ucMGP.

## Conclusions

The robust independent positive association between RRI and dp‐ucMGP suggests that RRI directly reflects the global atherosclerotic burden in a general population of healthy participants. This finding strengthens the potential role of RRI as a subclinical and prognostic marker of cardiovascular outcomes. Further prospective studies are needed to clarify whether MGP and RRI can be used as a stratification tool in the evaluation of individual cardiovascular risk.

It has already been shown that vitamin K supplementation dose‐dependently decreases dp‐ucMGP circulating levels and improves elastic properties of arterial vessel walls.^16^, ^33^, ^34^ Our findings suggest that an elevated RRI could identify patients benefiting from such supplementation. Whether this supplementation would result in a meaningful improvement in clinical outcome remains to be demonstrated in randomized controlled trials.

## Sources of Funding

The SKIPOGH (Swiss Kidney Project on Genes in Hypertension) study is supported by the Swiss National Science Foundation (FN 33CM30‐124087 and FN 33CM30‐140331). Belen Ponte was partially supported by the Swiss National Science Foundation (FN PMPDP3_171352).

## Disclosures

None.

## Supporting information


**Table S1.** Backward Stepwise Mixed Linear Regression (Without Outliers) for Renal Resistive Index on 970 Patients
**Table S2.** Factors Associated With Renal Resistive Index in Multivariate Mixed Linear Regression Models With Outliers but No Missing Covariates (n=1025)
**Figure S1.** Study flowchart.
**Figure S2.** Scatterplots showing the univariate association between renal resistive index, pulse pressure, and pulse wave velocity.
