# Supplementary material for: Renal Resistive Index Is Associated With Inactive Matrix Gla (γ‐Carboxyglutamate) Protein in an Adult Population‐Based Study
Source: J Am Heart Assoc. 2019 Sep 12;8(18):e013558. doi: 10.1161/JAHA.119.013558 (PMC6818003; doi:10.1161/JAHA.119.013558)
Supplement: Supplementary file 1 — Table S1. Backward Stepwise Mixed Linear Regression (Without Outliers) for Renal Resistive Index on 970 Patients Table S2. Factors Associated With Renal Resistive Index in Multivariate Mixed Linear Regression Models With Outliers but No Missing Covariates (n=1025) Figure S1. Study flowchart. Figure S2. Scatterplots showing the univariate association between renal resistive index, pulse pressure, and pulse wave velocity. [file JAH3-8-e013558-s001.pdf]

SUPPLEMENTAL MATERIAL

Table S1. Backward stepwise mixed linear regression (without outliers) for RRI on 970 patients.

Independent variables	Full model		Step 1		Step 2		Step 3		Step 4	
	β	p	β	p	β	p	β	p	β	p
Dp-ucMGP (nmol/l)	2.22	0.001	2.23	<0.001	2.18	0.001	2.11	0.001	2.18	0.001
Sex (female)	1.75	<0.001	1.76	<0.001	1.77	<0.001	1.73	<0.001	1.73	<0.001
Smoker (yes)	0.35	0.17	0.35	0.17	0.34	**0.18**				
Diabetes (yes)	0.67	0.32	0.67	**0.32**						
CV disease (yes)	0.55	0.14	0.55	0.14	0.57	0.12	0.56	**0.13**		
Age (years)	-0.16	<0.001	-0.16	<0.001	-0.16	<0.001	-0.16	<0.001	-0.16	<0.001
Age^2 ($years^2$)	0.003	<0.001	0.003	<0.001	0.003	<0.001	0.003	<0.001	0.003	<0.001
BMI (kg/m^2)	0.08	0.009	0.08	0.009	0.08	0.007	0.08	0.009	0.08	0.006
SBP (mmHg)	0.13	<0.001	0.13	<0.001	0.13	<0.001	0.13	<0.001	0.13	<0.001
DBP (mmHg)	-0.22	<0.001	-0.22	<0.001	-0.22	<0.001	-0.22	<0.001	-0.22	<0.001
Heart rate (1/min)	-0.05	<0.001	-0.05	<0.001	-0.05	<0.001	-0.05	<0.001	-0.05	<0.001
GFR ($ml/min/m^2$)	-0.001	**0.90**								
LDL (mmol/l)	-0.23	0.07	-0.23	0.07	-0.26	0.047	-0.26	0.041	-0.29	0.025

Glucose (mmol/l)	0.58	0.005	0.58	0.005	0.66	<0.001	0.66	<0.001	0.66	<0.001

All models adjusted for center as fixed effect.

RRI, renal resistive index; BP, blood pressure; CV, cardiovascular; CKD, chronic kidney disease; BMI, body mass index; SBP, systolic blood pressure; DBP, diastolic blood pressure; HR, heart rate; dp-ucMGP, dephospho-uncarboxylated matrix Gla protein; GFR, glomerular filtration rate; LDL, low dendity lipoprotein; HDL, high density lipoprotein; PWV, pulse wave velocity.

Definitions: Diabetes, reported or treated or fasting blood glucose level >7 mmol/l.

All models are adjusted for center as fixed effect and family as random effect. β corresponds to 1 unit increase.

Table S2. Factors associated with RRI in multivariate mixed linear regression models with outliers but no missing covariates (N=1'025).

Independent variables	Final model with extreme outliers		
	**β**	**95% CI**	**p**
Dp-ucMGP (nmol/l)	1.71	0.74; 2.67	0.001
Sex (women)	1.71	1.26; 2.15	<0.001
Age (years)	-0.15	-0.23; -0.08	<0.001
Age^2 (years)	0.003	0.002; 0.004	<0.001
BMI (kg/m^2)	0.09	0.04; 0.15	0.001
SBP (mmHg)	0.14	0.11; 0.16	<0.001
DBP (mmHg)	-0.24	-0.28; -0.20	<0.001
HR (1/min)	-0.06	-0.08; -0.03	<0.001
LDL (mmol/l)	-0.26	-0.50; -0.01	0.041
Glucose (mmol/l)	0.71	0.36; 1.07	<0.001

RRI, renal resistive index; BMI, body mass index; SBP, systolic blood pressure; DBP, diastolic blood pressure; HR, heart rate; dp-ucMGP, dephospho-uncarboxylated matrix Gla protein; LDL, low density lipoprotein.

Figure S1. Study flowchart.

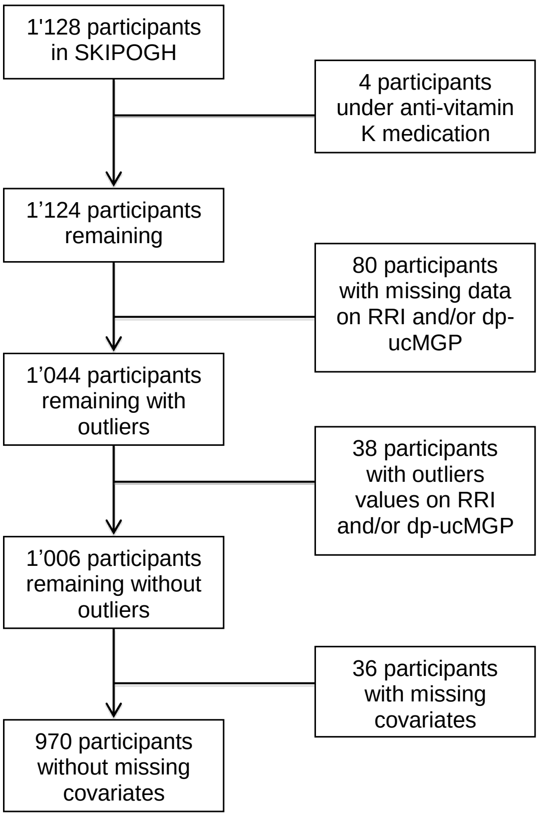

RRI, renal resistive index; dp-ucMGP, dephospho-uncarboxylated matrix Gla protein

Figure S2. Scatterplots showing the univariate association between RRI, PP and PWV.

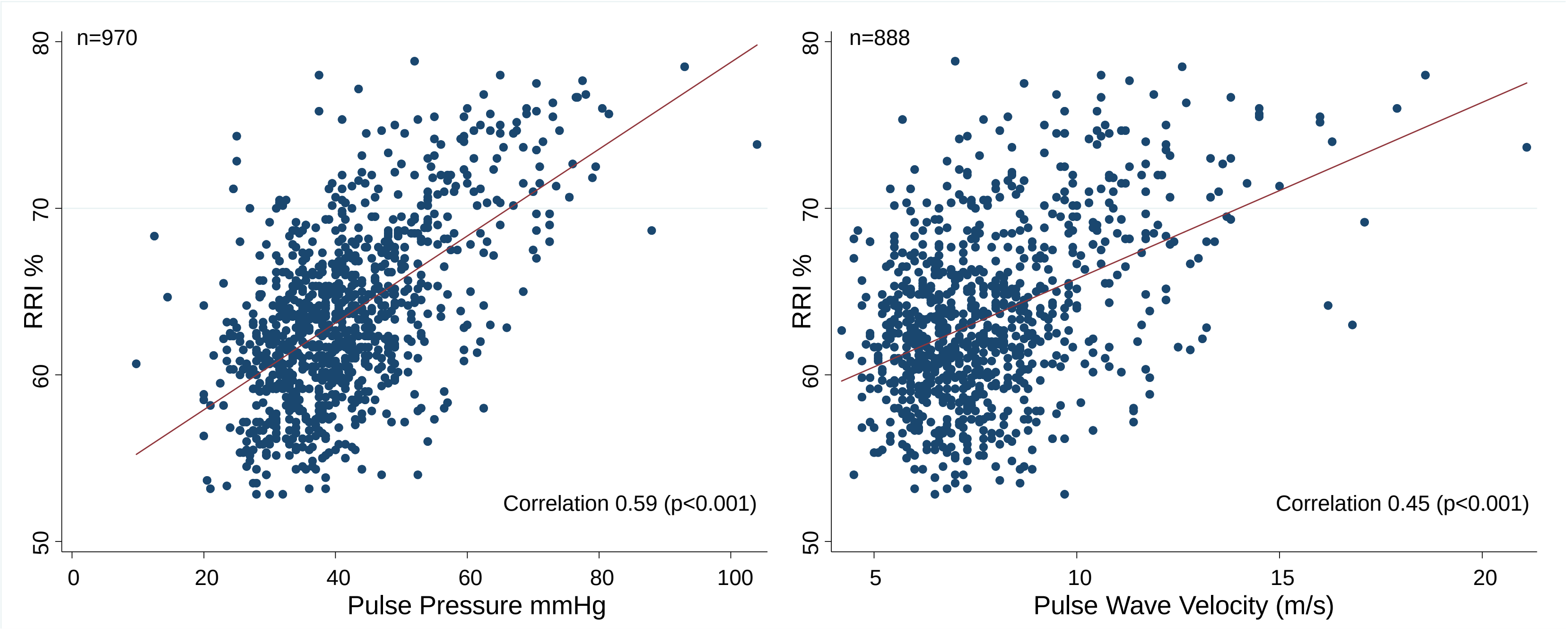

a. Renal Resistive Index and Pulse Pressure

b. Renal Resistive Index and Pulse Wave Velocity

Pearson correlation coefficients are represented in the graph with the corresponding p-value.

RRI, renal resistive index; PP, pulse pressure; PWV, pulse wave velocity.
